# Measurement data from full-scale fire experiments of battery electric vehicles and internal combustion engine vehicles

**DOI:** 10.1016/j.dib.2026.112471

**Published:** 2026-01-16

**Authors:** Nathaniel G. Sauer, Matthew J. DiDomizio, Richard M. Kesler, Shruti Ghanekar, Parham Dehghani, Gavin P. Horn, Adam Barowy

**Affiliations:** UL Research Institutes, Fire Safety Research Institute, 6200 Old Dobbin Lane, Suite 150, Columbia, MD 21045, United States

**Keywords:** Electric vehicle, Automobile, Lithium-ion battery, Combustion, Temperature, Heat transfer, Gas composition, Chemical exposure

## Abstract

Over time, the burning behaviour of passenger vehicles has been influenced by continuous advancements in vehicle design, including changes in materials, manufacturing processes, and the integration of modern lithium-ion battery powertrains. Fire protection engineers, first responders, and other safety professionals currently lack sufficient data to determine whether existing fire protection system designs and firefighting practices effectively mitigate the burning behaviour of modern vehicle fires or to adapt these approaches to any measurable changes in fire dynamics. Eighteen full-scale vehicle fire experiments were conducted in a laboratory environment to obtain a novel data set describing the fire size, thermal hazards, and evolved smoke and particulate species resulting from vehicle fires. Vehicle mass, gas temperature, heat flux, sorbent tube, Fourier-transform infrared spectrometer, and suppression waterflow data are provided in tabular format. Eight vehicle models across six manufacturers were selected based on the most popular battery electric and gasoline powered compact and mid-sized recently sold in North America at the time of writing. Experiments consisted of nine free-burn experiments wherein the gasoline vehicles were ignited in the engine compartment and the electric vehicle battery packs were induced into thermal runaway; these fires progressed unabated until the occurrence of natural flame extinction. Another four experiments simulated electric vehicle fire suppression using ordinary water and traditional vehicle fire suppression techniques. In one additional experiment, an encapsulator firefighting agent was added to the water. The final four experiments consisted of deploying fire blankets over the burning vehicles a single strategy or in combination with water simultaneously applied from beneath. There are many potential uses of this data, but primary uses are expected to include revision of design criteria and guidance within the fire protection engineering community, strategic and tactical decision aids for vehicle fire incident operations, validation of existing (and development of new) fire behavior models, and guidance to vehicle manufacturers for improved fire safety design.

Specifications Table

**Table:** 

Subject	Engineering & Materials science
Specific subject area	Behaviour of modern lithium-ion battery and gasoline powered vehicle fires and effectiveness of traditional and emerging firefighting approaches.
Type of data	Table (.csv and .pdf format) raw and processed
Data collection	Vehicles were ignited from beneath using a 30 kW propane burner. Air samples were collected with personal air sampling pumps using sorbent tubes and/or filters to quantify masses of vapors and particulates. Heat flux was measured by Schmidt-Boelter and plate heat flux sensors. Temperatures in the cabin and on/inside the battery were measured with type-K thermocouples. A system of weighing platforms provided vehicle mass loss measurements. An extractive FTIR was used to identify chemical species in the smoke plume. Suppression water flowrate was measured with a turbine mass flowmeter.
Data source location	• Institution: UL Research Institutes • City/Town/Region: Northbrook, Illinois • Country: United States • Latitude and longitude for collected samples/data: 42.14616, -87.84650
Data accessibility	Repository name: Measurement data from full-scale fire experiments of battery electric vehicles and internal combustion engine vehicles Data identification number: 10.60752/102376.30438392 Direct URL to data: https://doi.org/10.60752/102376.30438392.v1
Related research article	None

## 1 Value of the Data

• These data address the scarcity of full-scale fire measurements available to quantify the burning behavior of modern North American passenger vehicles with internal combustion engine and lithium-ion battery electric powertrains. Most available data prior to these experiments was collected from vehicle makes and models that are 20 to 40 years old, Whereas modern vehicles incorporate substantially different materials including increased synthetics known to influence fire dynamics. Such comparison data are scarce as the fire sizes are too large to be safely studied in most fire laboratories and are cost-prohibitive.
• This data will be of particular utility to fire safety engineering professionals, fire model developers and practitioners, fire investigators, fire code and policy developers, authorities having jurisdiction, vehicle manufacturers and risk modelers among others.
• Measurements included in this dataset include the thermal exposures from vehicle fires to targets surrounding the vehicle (temporally and spatially varying heat flux measurements). These measurements enable analyses of risk to life safety, fire spread, and property damage. Furthermore, mass loss rate and gas temperature measurements provide an understanding of the rate of fire growth, peak fire size, fire duration, and fire position with time.
• Chemical exposure health hazard measurements are included in these data, which may be used to inform and develop updated occupational safety practices for first responders during firefighting, and for second responders during fire investigation, incident cleanup, and vehicle salvage and recycling.
• There is significant value in using these data to update building and fire protection system design inputs and assumptions, to validate vehicle fire heat release rate models, and to generalize thermal exposure risks to adjacent combustible exposures.
• These data include the only known publicly accessible measurements that can be used to evaluate the effectiveness of electric vehicle fire suppression with water from fire hoses, specialized EV-specific water spray appliances, and the application of high-temperature textile blankets intended for controlling the combustion of ordinary combustible materials used in vehicle chassis construction.

## 2 Background

This dataset was developed to contribute to the global need to distinguish how vehicle fire behavior is changing with evolving construction materials and designs, and the transition from gasoline to lithium-ion battery propulsion systems. This dataset is intended to be of direct utility to firefighters, fire protection engineers (FPEs), and fire researchers; additionally, authorities having jurisdiction, insurance professionals, and building owners are anticipated to benefit from access to these data.

Operational experience in electric vehicle fire incident response has demonstrated that firefighter safety and firefighting efficacy can be substantially improved by data that informs the development of improved vehicle fire standard operating procedures as well as real-time tactical decision making. Data that support these developments includes time-history of fire development, incident radiative heat flux, characterization of air and water runoff chemical species contamination, the timing and flowrate of firefighting hose streams, and the timing of vehicle fire blanket deployment.

FPEs must understand the characteristics of fire development to properly design life safety and building fire protection systems for mitigating modern vehicle fire hazards. Data of interest include fire growth rate, peak fire size and duration, and thermal exposures to adjacent vehicles or structures; these performance metrics may be determined from the measured time histories of mass loss rate and incident radiative heat flux.

To illustrate how these data may be applied, suppression intervention timelines and temperature data during firefighting operations can be used to support quantitative evaluation of firefighter tactics, hose stream deployment, and the effectiveness of suppression and cooling strategies for both internal combustion engine and electric vehicles. This could aid fire departments in incident planning, knowing the effectiveness and options available when addressing a vehicle fire. Chemical species measurements in air and water runoff support evaluation of responder exposure hazards and post-incident environmental contamination. Thermal exposure data may be used by fire protection engineers and vehicle designers to assess heat transfer to adjacent vehicles or structures, validate vehicle fire and suppression models, and evaluate the performance of passive and active fire protection measures intended to limit fire spread or reduce structural impact during vehicle fire incidents.

## 3 Data Description

The root directory of the data repository [1] contains five files and 18 directories (one per experiment). The contents of each directory are detailed in Table 1. The name of each directory corresponds to the experiment ID (see Table 2). In the root directory, the file readme.md (also in .pdf form) contains information about the data repository. The file instrumentation.csv details the changes in instrument configurations and locations across all experiments. The file instrumentation.pdf is an illustration which shows laboratory setup and instrumentation positions. The file vehicle_info.csv contains information on the vehicles used in each experiment.

**Table 1:** Contents of each experiment directory in the data repository.

File name	Description of file
information.md	A plain-text file containing information about the experiment, including details of the vehicle and initiating fire.
events.csv	A comma-separated value file (CSV) in which the first column contains the description of an event and the second column contains the corresponding time of the event (format hh:mm:ss). Time series data has been shifted relative to ignition, where ignition is at time zero (t = 0 s).
data_timeseries.csv	A CSV file in which the first column contains a time stamp (shifted relative to ignition of the initiating fire) and each other column contains a measurement at the corresponding time. This file includes measurements from heat flux gauges, thermocouples, flow meters, water additive load cell, and mass flow controller.
data_massloss.csv	A CSV file in which the first column contains a time stamp (shifted relative to ignition of the initiating fire), columns 2 through 5 contain the output of each of the four platform scales measuring the mass of the vehicle, column 6 contains the total mass of the vehicle, column 7 contains the calculated mass loss rate, and column 8 contains the calculated heat release rate.
data_heatflux.zip	A zip directory containing 12 files in hierarchical data format (HDF5), which may be read using the h5py Python package. These files contain measurements of the temperature and incident radiative heat flux over the surface of the plate sensors. Six files are named “T_XX” (temperature) and six files are named “HF_XX” (incident radiative heat flux), where “XX” in the file name indicates the position of the panel: DF = driver-front; DM = driver-middle, DR = driver-rear, PF = passenger-front, PM = passenger-middle, and PR = passenger-rear.
data_ftir.zip	A zip file containing FTIR data. Each zip file contains: a) a plain text file named ‘Info-file.txt’ containing details of the FTIR parameters; b) a CSV file containing a log of the gas cell pressure in hectopascal (hPa) and temperature in Kelvin (K) starting at burner ignition; and c) a directory named ‘Spectra’ containing a series of CSV files where the first column is the wavenumber in inverse centimeters (cm^-1^) and the second column is the transmitted intensity. The name of the CSV file follows the format: <data point after burner ignition>_(<time stamp>). The time stamp follows the format: yyyy_mm_dd_hr_mm_ss_sss, where sss is milliseconds.
Cabin Fluoride Filter Results.pdf	A portable document file (PDF) that contains analytical results of quantification of particulate fluoride and hydrofluoric acid in sample collected using fluoride filter from the cabin of the vehicle during the experiment.
data_airsampling.csv	A CSV file in which the first column indicates the test name and the second column indicates the sampling location. The first row of columns 3 through 47 include the compound that was measured and the unit of measure. Rows 2 through 4 include the mass of each compound that was collected corresponding to the header in row 1. If the mass was below the level of detection (LOD) the mass is listed as ‘<LOD’. If a sample was not collected for a compound or location, that mass is listed as ‘NA’.
data_airsamplingtime.csv	A CSV in which the first column indicates the test name and the second column indicates the sampling location. Each row contains the time (in minutes) that each sampling pump ran, corresponding to the header in row 1. If a sample was not collected for a compound or location, that time is listed as ‘NA’.

**Table 2:** Information on each of the experiments included in the data repository.

Experiment ID	Vehicle Type	Vehicle Make & Model	Experiment Type
G-HK-F	ICEV	Hyundai Kona SEL	Free burn
E-CB-F	BEV	Chevy Bolt EV LT	Free burn
E-NL-F	BEV	Nissan Leaf S	Free burn
E-HI-F	BEV	Hyundai Ioniq Limited	Free burn
E-TM-F	BEV	Tesla Model 3 Long Range	Free burn
E-FM-F	BEV	Ford Mustang Mach E GT	Free burn
E-HK-F	BEV	Hyundai Kona SEL	Free burn
G-HK-F2	ICEV	Hyundai Kona SEL	Free burn
G-TR-F	ICEV	Toyota RAV4 XLE AWD	Free burn
E-HK-S	BEV	Hyundai Kona SEL	Water suppression
E-CB-S	BEV	Chevy Bolt EV LT	Water suppression
E-TM-S	BEV	Tesla Model 3 Long Range	Water suppression
E-FM-S	BEV	Ford Mustang Mach E GT	Water suppression
E-FM-SA	BEV	Ford Mustang Mach E GT	Water suppression with encapsulator agent
E-FM-B	BEV	Ford Mustang Mach E GT	Fire blanket
E-TM-B	BEV	Tesla Model 3 Long Range	Fire blanket
E-FM-BS	BEV	Ford Mustang Mach E GT	Fire blanket with water suppression
E-TM-BS	BEV	Tesla Model 3 Long Range	Fire blanket with water suppression

## 4 Experimental Design, Materials and Methods

### 4.1 Experiment and vehicle types

A total of 18 experiments were performed, consisting of 9 free burn experiments (in which the vehicle was allowed to burn to completion without any intervention), 5 suppression experiments (in which water or water with a encapsulator agent was employed to suppress the fire), and 4 fire blanket experiments (in which a fire blanket, in some cases in combination with water suppression, was employed to suppress the fire). The vehicles used in these experiments included 3 internal combustion engine vehicles and 15 battery electric vehicles. The vehicle make and model used in each experiment are provided in Table 2; additional details are provided in the data repository. The most popular electric vehicles by total United States sales volume were selected for testing, with the addition of the Hyundai Kona as the only North American vehicle that used the same chassis for its battery electric vehicle (BEV) and internal combustion engine (ICEV) chassis.

### 4.2 Laboratory setup

**Fig. 1:**
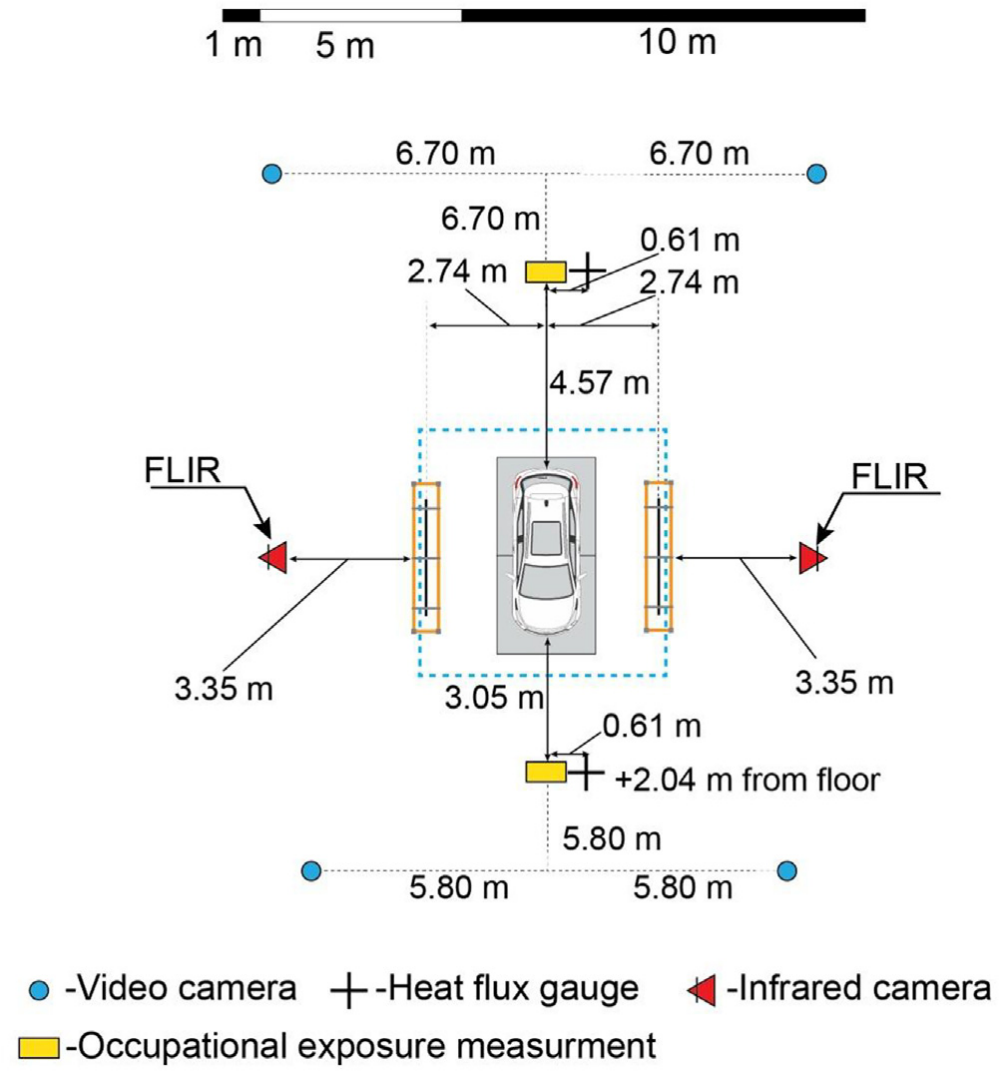
Plan view of the laboratory space in which experiments were conducted (to scale).

All experiments were performed in an indoor laboratory space to eliminate the influence of outdoor wind conditions. The laboratory space had a footprint of 33 m by 33 m. The vehicles were centered under a movable ceiling that measured 30 m by 30 m and was positioned 9.1 m above grade. An exhaust duct was located above the movable ceiling, and a flow rate of 28.3 m^3^ s^-1^ was maintained throughout each experiment to extract fire effluent from the laboratory space. Vehicles were positioned inside a steel pan having inside dimensions of 2.44 m wide by 4.88 m long, with a lip height of 0.17 m. The pan was levelled on four platform scales (described below) such that the lip was 0.56 m above grade. The inside of the pan was lined with 1.3 cm thick DensDeck fiberglass mat-faced, gypsum core panels to thermally protect the pan and underlying platform scales. Fig. 1 shows the instrumentation layout used for a majority of the free burn experiments (E-CB-F through G-TR-F); any deviations from this layout are described in the data repository.

### 4.3 Experimental procedure

**Fig. 2:**
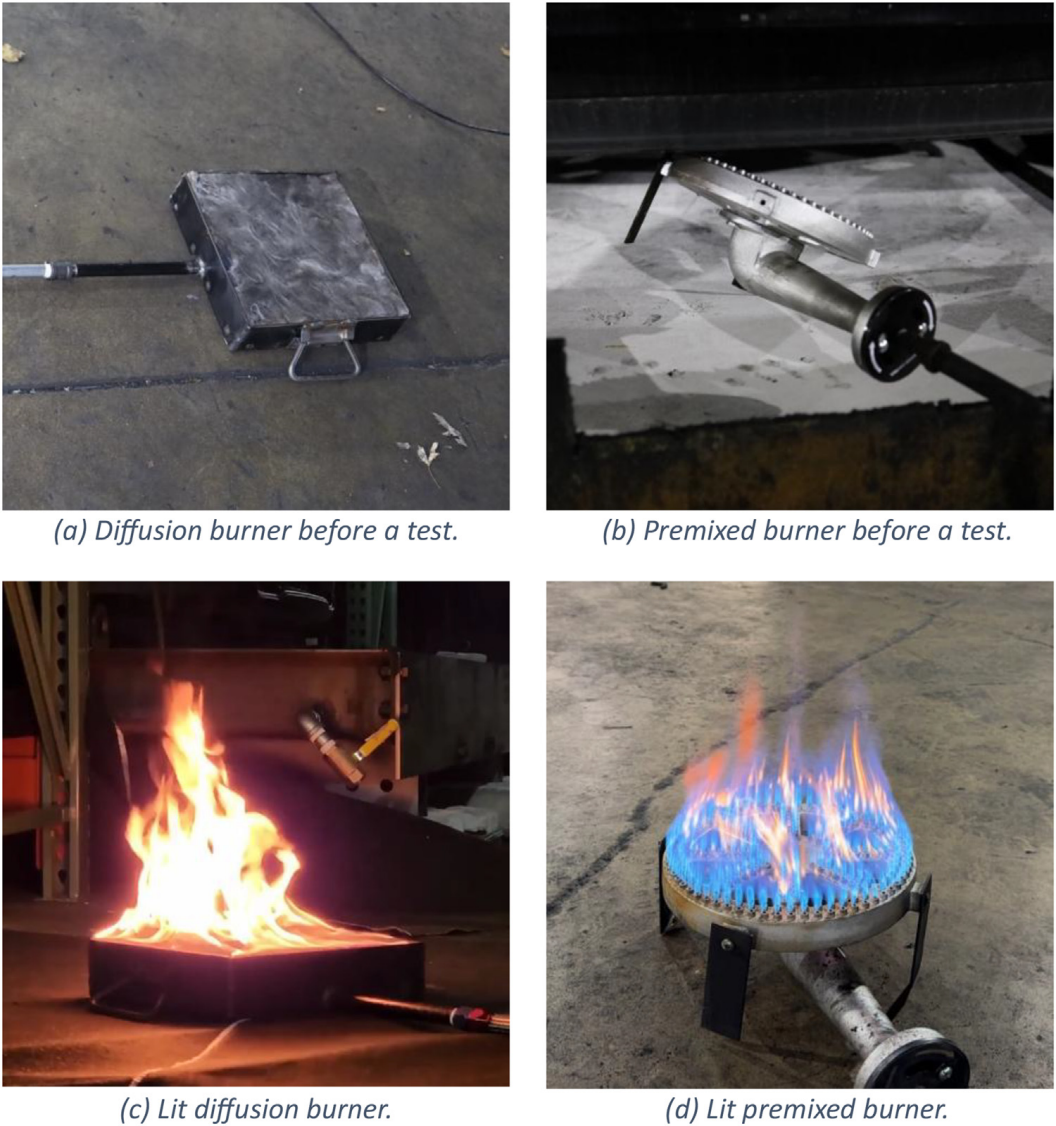
Diffusion and premixed burners used to initiate the vehicle fires.

Prior to each BEV experiment, the vehicle was charged to its maximum state of charge. For both BEV and ICEV experiments, windows were set to their lowest positions, and the vehicles were set to their “ON” positions. A burner was positioned under the vehicle to initiate each fire. Two burner types were used: a propane diffusion burner (shown in Fig. 2a and c) and a pre-mixed propane and air burner (shown in Fig. 2b and d). The burners were configured to deliver an initial heat release rate of 30 kW (corresponding to 21 SLPM of propane). The burner used for each experiment is provided in the data repository.

Experiments were initiated by simultaneously starting all data acquisition systems (detailed in the following section). After collecting two minutes of background data, ignition of the burner was initiated. The occurrence of ignition and all other observations of note were recorded in an events file for each experiment. Battery temperatures were monitored in the initial heating phase of the experiments. If temperatures stabilized and fire was not observed to spread to involve either the cabin or battery pack, the burner output was increased by 5 kW. This procedure was repeated until the occurrence of thermal runaway or fire spread to the cabin, whichever occurred first, at which time gas flow to the burner was terminated.

For the free burn experiments, the fire was allowed to spread to involve the entire vehicle. With one exception, no attempt at suppression or other intervention was made, and the experiments were continued until fire extinction. In one instance (E-TM-F) the effluent overcame the building’s exhaust capacity, and the test was terminated by water suppression.

**Fig. 3:**
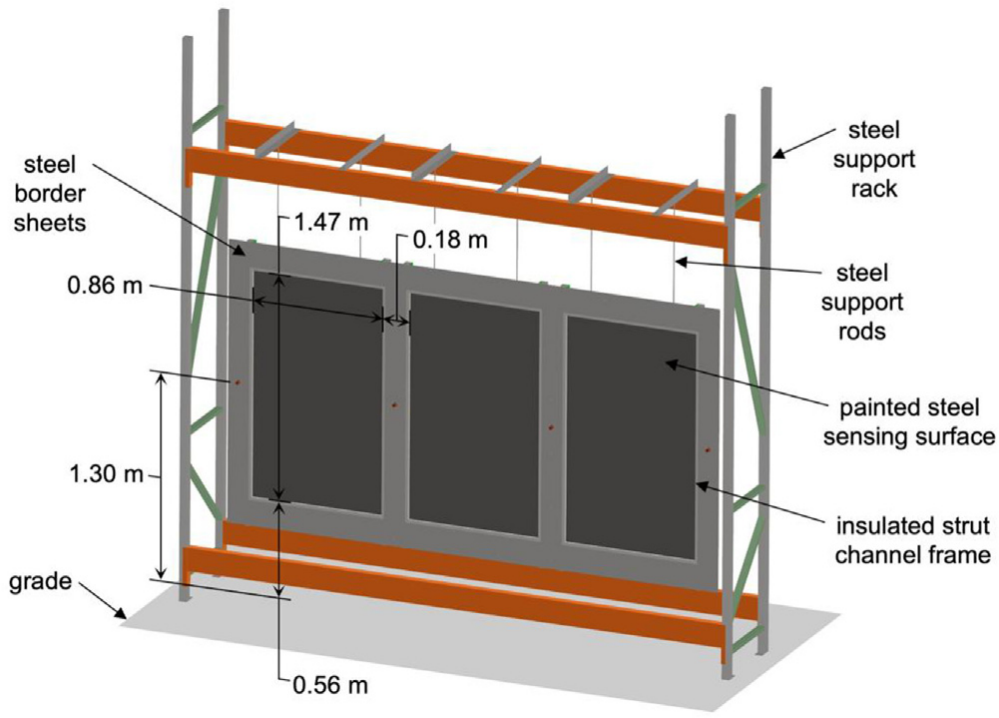
Three-dimensional rendering of a plate sensor array.

For the water suppression experiments, the fire was allowed to spread following cabin ignition or thermal runaway for a period of 6 minutes to replicate the standard response time goal objective for North American emergency services [2]. After 6 minutes, staff entered the laboratory, prepared fire suppression equipment, and began fire suppression operations. Fire suppression operations involved spraying water into the burning vehicle cabin and on the burning vehicle exterior until visible flaming was extinguished. When the battery was involved in the fire, flames continued emanating from the bottom of the vehicles. Suppression operations continued for 10 minutes until a change in firefighting tactics was simulated; water flow was stopped, and the vehicle was tilted using powered equipment to provide more direct hose stream access to the battery pack. From this point, suppression operations continued until all visible flaming ceased. Four of the suppression experiments utilized solely water, but for the E-FM-SA experiment, water with an encapsulator agent was used. Water was applied with the standard firefighting combination nozzle shown in Fig. 3a with 3.8 cm (1.5 in) inlet, designed to flow 567.8 L min^-1^ (150 gal min^-1^) at 3.45 bar (50 psi) nozzle pressure (ChiefXD, Elkhart Brass, Elkhart, IN). When used, the encapsulator agent was introduced with an eductor at a concentration of approximately 3 % v/v, but the procedure was otherwise the same as suppression with water.

For the two fire blanket experiments without suppression, fire blankets were deployed after a simulated 6-minute emergency response arrival time. The fire blankets were deployed using procedures recommended by the manufacturers; the blanket was positioned in front of the vehicle, unrolled/unfolded to a ready position, and firefighters pulled opposite corners on the same end of the blanket over the burning vehicle, from the front to the rear in one continuous motion. Once the vehicle was fully covered, the blanket was tucked under the base of the vehicle, smoothing the excess material and minimizing wrinkles as much as possible. As firefighters repositioned safely away from the vehicle, a cable and pulley were quickly attached to allow lifting one corner of the blanket remotely. This enabled lifting a small area of the blanket to investigate the sensitivity of vehicle and accumulated flammable gas re-ignition when introducing ambient air to simulate inspecting fire conditions or insertion of under-vehicle nozzles. The blanket was lifted several times from minutes to tens of minutes after its deployment.

For the two fire blanket experiments with water suppression, the vehicle fires were initially suppressed with the firefighting nozzle following the timing and procedure used in the water suppression experiments. Once the vehicle cabin fire was suppressed, the fire blanket was deployed using the same procedure as the fire blanket experiments. The blanket was initially lifted to simulate positioning of an EV-specific upward-spraying nozzle (Transformer Nozzle System, Task Force Tips, Valparaiso, IN) beneath the vehicle battery pack. Shortly thereafter, water flow was initiated through this nozzle and continued for several minutes. The nozzle was operated at the manufacturer specified 6.9 bar (100 psi) to supply a claimed flow of 567.8 L min^-1^ (150 gal min^-1^). Nozzle flow was started and stopped, and the blanket was partially lifted multiple times to investigate impact on battery cooling and smoke flammability, respectively.

Upon the conclusion of each experiment, data acquisition systems were terminated, and data files were backed up to external media. Post-experiment photographs of the vehicle were obtained to document its condition and the fire involvement.

### 4.4 Measurement apparatus

#### 4.4.1 Ignition burner

The propane diffusion burner was 7.6 cm tall, with a square opening having side lengths of 30.5 cm. A steel mesh supported by an internal structure created a plenum space into which propane was supplied. Cerablanket 8 PCF ceramic fiber insulation was sandwiched between layers of steel mesh to provide flow-resistance and a uniform surface from which the propane was uniformly emitted. Above the steel mesh the fuel mixed with ambient air, forming a diffusion flame. Propane was supplied to the burner from a tank, and the supply flow rate was controlled with an Alicat 50 SLPM (MCRS-50SLPM-D-20 × 32/5M) mass flow controller.

The premixed propane and air tube burner consisted of a fuel inlet and a circular emitting surface having an overall diameter of 30.5 cm and height of 12 cm. The emitting surface contained approximately 224 holes, each having a diameter of about 4 mm. The burner was supplied with a mixture of propane and air. The propane supply was the same as that used for the diffusion burner. Dry air was added to the propane with a mixing valve from an air source regulated to the same pressure as the propane supply. The flow rate of air was regulated with a needle valve to achieve a similar heat output to the diffusion burner, but with smaller and more intense flames. The heat output of the premixed burner may have varied slightly from that of the diffusion burner due to combustion efficiency, but was still nominally 30 kW for a propane supply rate of 21 SLPM.

#### 4.4.2 Vehicle mass

For the free burn experiments, the combined mass of the vehicle and pan were measured over time using four platform scales (one per corner of the pan). This approach to measuring the total vehicle mass is consistent with that adopted by researchers in previous experimental studies on vehicle fires [3]. Each scale (Mettler-Toledo model PFD779 US11 with a IND570 indicator) had a capacity of 1500 kg, resolution of 100 g, and repeatability of 90 g. The mass of the vehicle was not measured for suppression or fire blanket experiments due to the short burning durations in those experiments.

Computer recording of the mass data for experiment HKG-F2 failed, and for experiment TRG-F mass recording failed partially during the later portions of the experiment. Video recordings of the digital readouts of the indicators were obtained for the HKG-F2 experiment, and these readouts were manually digitalized at 4 second increments until the indicator screens became too obscured with smoke to read. Attempts were made to digitize the lost TRG-F data, but the platform scales experienced failure during portions of the experiments when the digital readouts were impossible to read. Mass data for these experiments during periods of measurement failure has been omitted from the data repository.

#### 4.4.3 Heat flux

Heat flux to the surroundings of the vehicle was measured using water-cooled Schmidt-Boelter heat flux gauges and plate sensors.

For the free burn experiments, plate sensors were used to measure the spatially and temporally varying heat flux to both the driver and passenger sides of the vehicle. These sensors were constructed in accordance with the guidance set forth by DiDomizio and Dehghani [4]. Each plate sensor included a sensing surface of 0.79 mm thick (22 ga) 304 stainless steel sheet coated in high-temperature high-emissivity black paint (Rust-Oleum Specialty High Heat having an emissivity of 0.94). The sensing surface was insulated on all four edges with 2.5 cm of Cerablanket 8 PCF ceramic fiber insulation and framed in 4.1 cm galvanized steel strut channel. The exposed area of the sensing surface measured 86.4 cm wide by 147.3 cm tall. Plate sensor arrays were constructed by arranging three plate sensors side-by-side. Each plate sensor was suspended from a steel support rack using two threaded steel rods, which were bolted to the plate sensor frame and to the support rack. Plate sensors were positioned such that the distance between the edges of the adjacent sensing surfaces was 17.8 cm, and the center of each sensor was 129.5 cm above grade. Sheets of 0.79 mm thick (22 ga) carbon steel were fastened to the sides, tops, and bottoms of the framing of each plate sensor to prevent airflow between them. The total size of the heat flux sensor arrays measured 3.31 m wide by 1.84 m tall. Fig. 3 shows a three-dimensional rendering of the plate sensor arrays.

**Fig. 4:**
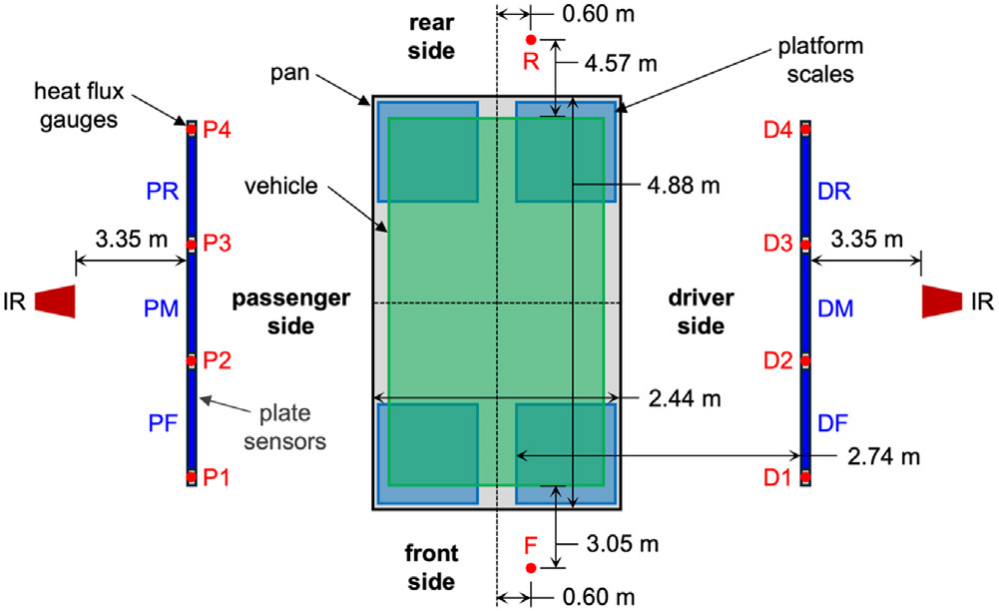
Plan view of plate sensor and heat flux gauge positions (not to scale).

Plate sensor arrays were positioned parallel to both the passenger and driver sides of the vehicle, as depicted in Fig. 4. The distance between the sensing surface of the plate sensors and the center-plane of the vehicle was 1.83 m for the G-HK-F experiment, and 2.74 m for all other free burn experiments. Each panel was assigned a unique ID, as shown in the Fig., based on its position relative to the vehicle (e.g., the DR panel was at the driver-side rear position, while the PF panel was at the passenger-side front position). These same IDs are used in corresponding file names in the data repository.

FLIR A70 infrared (IR) cameras were positioned on the unexposed sides of both the driver-side and passenger-side plate sensor array, aligned with the mid-point of each array. The IR cameras had uncooled microbolometer sensors with a spectral range of 7.5 µm to 14 µm, resolution of 640 px wide by 480 px tall, and field of view of 51°. At a separation distance of 3.35 m from the plate sensors, the projected pixel dimension was approximately 6.3 mm. Thermogram sequences were recorded of each plate sensor array.

Each plate sensor array incorporated four Medtherm 64-series Schmidt-Boelter water cooled heat flux gauges. These gauges were aligned with the mid-height of the plate sensors, 1.30 m above grade, and mounted flush to the carbon steel plates between the plate sensors. The heat flux gauge IDs indicated in Fig. 4 correspond to those used in the data repository. The central two gauges on each side (D2, D3, P2, and P3) were combination total heat flux and windowed radiometer sensors. The radiometer sensors had sapphire windows with a viewing angle of 150°. The outer two gauges on each side (D1, D4, P1, and P4) were windowed radiometer sensors, having sapphire windows with a viewing angle of 150°. All eight heat flux gauges on the driver and passenger sides of the vehicle were cooled with water supplied at 30°C throughout all experiments.

In addition to the heat flux gauges on the driver and passenger sides, total heat flux gauges were positioned 3.0 m from the front bumper and 4.5 m from the rear bumper of the vehicle, offset from the vehicle’s longitudinal plane by 0.6 m toward the driver side (to accommodate the air sampling stands). Each of these sensors was positioned 2.0 m above grade. The front and rear heat flux gauges were cooled with water supplied at 10°C throughout all experiments.

Neither plate sensors nor driver-side heat flux gauges were present for the suppression or fire blanket experiments, as they would interfere with firefighting operations. The four passenger-side heat flux gauges remained and were affixed to steel stands at the same positions as those used in the free burn experiments.

#### 4.4.4 Temperature

Gas temperatures within the vehicles, at the front and rear heat flux gauge locations, above the vehicle in the plum, and within the battery packs (for BEV experiments) were measured using thermocouples (TCs).

**Fig. 5:**
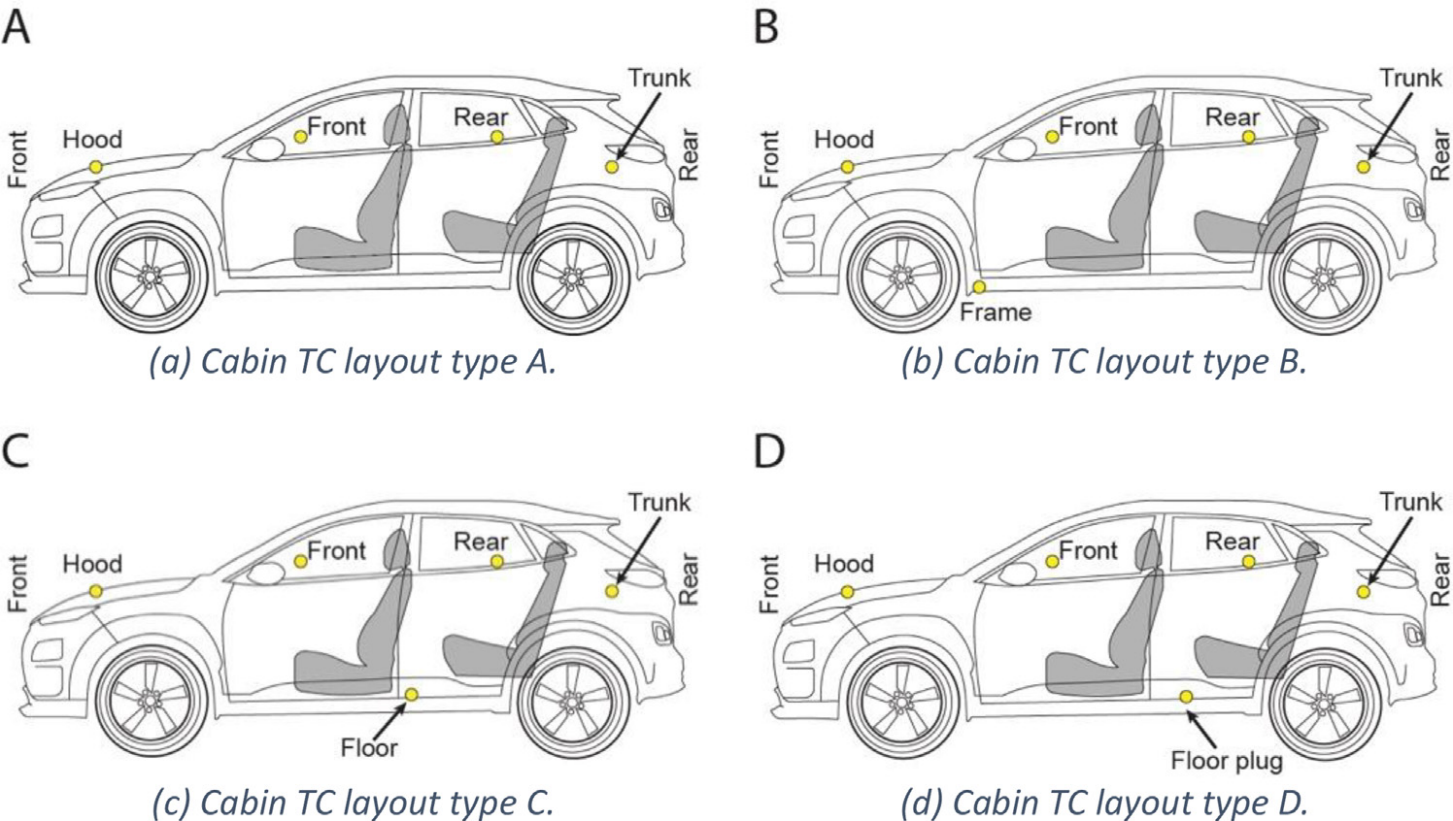
Profile views of cabin thermocouple locations and layout types referenced in Table 3.

Thermocouples inside the vehicle cabin were installed at approximately passenger head-height. Thermocouples in the hood, front seat, rear seat, and trunk were present for all tests. Thermocouples were added to penetrations in the floor of the passenger cabin when applicable. These changes per-test are noted in Table 3 and Fig. 5. Passenger cabin thermocouples were secured with steel wire and self-tapping screws.

**Table 3:** Car thermocouple layout figure key.

Code	Make	Model	Cabin TC layout	Battery TC layout
G-HK-F	Hyundai	Kona SEL	B	N/A
E-CB-F	Chevy	Bolt EV LT	A	A
E-NL-F	Nissan	Leaf S	A	B
E-HI-F	Hyundai	Ioniq Limited	C	E
E-TM-F	Tesla	Model 3 Long Range	C	D
E-FM-F	Ford	Mustang Mach E GT	C	A
E-HK-F	Hyundai	Kona SEL	C	C
G-HK-F2	Hyundai	Kona SEL	A	N/A
G-TR-F	Toyota	RAV4	A	N/A
E-HK-S	Hyundai	Kona SEL	A	A
E-CB-S	Chevy	Bolt EV LT	A	A
E-TM-S	Tesla	Model 3	A	D
E-FM-S	Ford	Mustang Mach E	A	F
E-FM-B	Ford	Mustang Mach E	A	G
E-TM-B	Tesla	Model 3	A	H
E-FM-SA	Ford	Mustang Mach E	A	G
E-FM-BS	Ford	Mustang Mach E	A	G
E-TM-BS	Tesla	Model 3	A	H

**Fig. 6:**
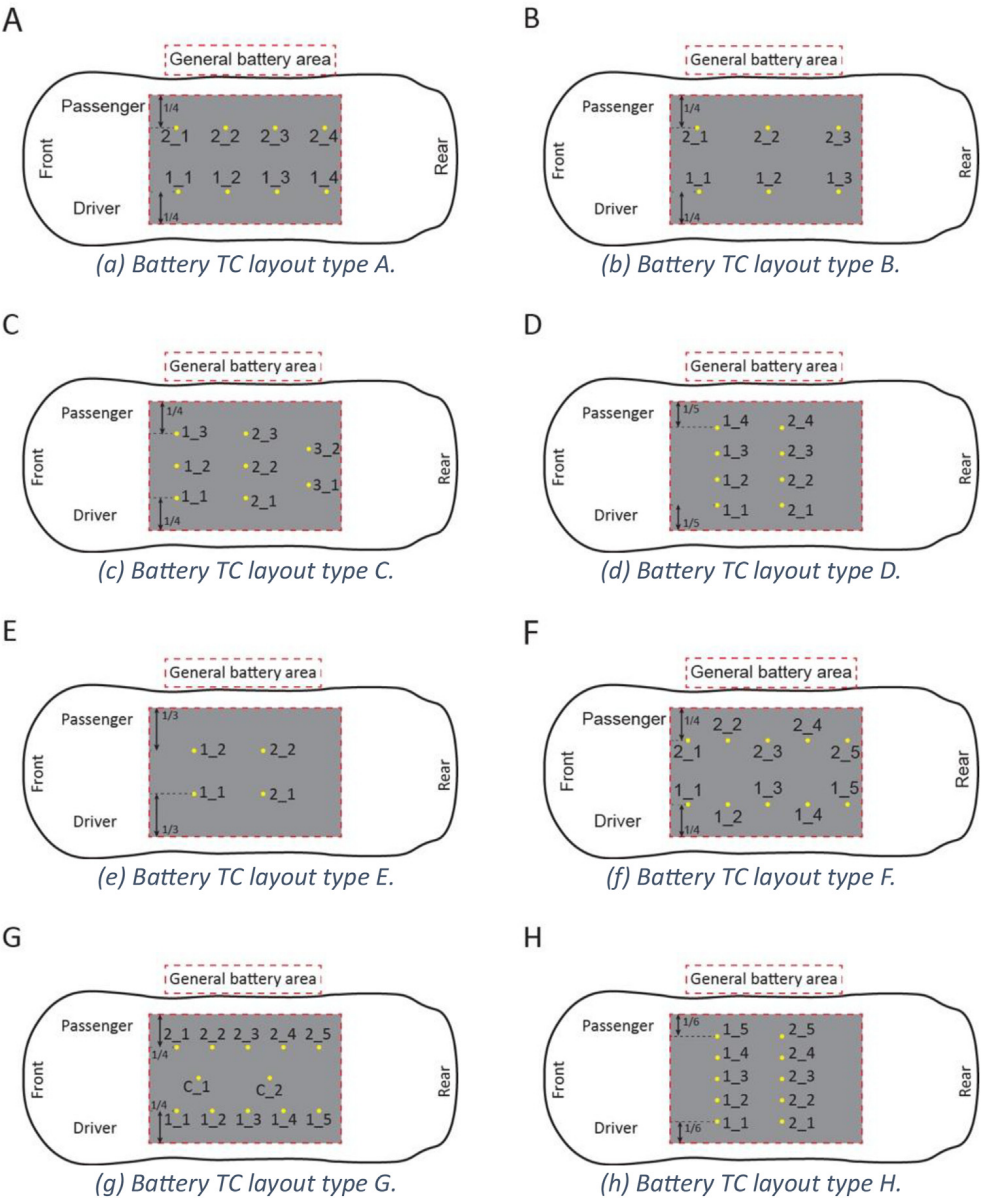
Plan views of EV battery thermocouple locations and layout types referenced in Table 3.

All electric vehicles had thermocouples placed in or on the bottom exterior of their battery packs to measure battery pack temperature over the duration of the experiment. For experiments E-CB-F through E-HK-F the battery thermocouples were surface mounted to the underside of the battery case using self-tapping screws and a type-K thermocouple crimped into a ring terminal connector. For experiments E-HK-S through E-FM-S shielded thermocouples were inserted between the bottom plate protective plate and internal cold plate of the battery pack through a small hole drilled in the bottom of the pack. In the last experiments E-FM-B through E-TM-BS thermocouples were placed on top of the battery modules inside of the pack and held in place with polyimide tape. To accomplish this the packs were partially disassembled, thermocouples located and secured, and then the packs reassembled. Battery thermocouple layouts are noted in Table 3 and Fig. 6.

### 4.5 Water flow rate

The volume flow rate of suppression water was measured using a ModMAG M2000 DN65 electromagnetic flowmeter with a range of 0.4 to 143 m^3^h^-1^. A Task Force Tips Eductor (UE-125-NF) was used to introduce extinguishing agent into the suppression water flow for a single experiment. An S-shaped 450 kg capacity load cell with an accuracy of ± 90 g was used to determine time-resolved usage of extinguishing agents.

### 4.6 Smoke species composition

A Fourier Transform Infrared (FTIR) Spectrometer equipped with a 2 m multi-reflection gas cell (Bruker Scientific MATRIX-MG2) was used to measure the composition of gas in the smoke from the vehicle. The gas cell has a volume of 200 mL and was fitted with ZnSe windows and gold coated mirrors with 6 mm compression fittings. During operation, the gas cell was heated to 180°C to mitigate condensation inside of the gas cell. The spectrometer was equipped with a liquid nitrogen-cooled MCT detector having a spectral resolution better than 0.5 cm^-1^ and a scan rate of up to 5 scans per second.

The probe locations, sampling intervals, and sample line lengths for the various vehicles are listed in Table 4. The sampling probe was a 9.5 mm (3/8 in.) C276 nickel alloy tube. The gas sampled from the probe was transported to the FTIR using a PTFE-lined heated sampling line maintained at 110°C throughout the experiment. The gas was passed through a coalescing filter (PermaPure FF-250-SS-2.5G) to remove particulates and condensate. The gas flow was maintained using a diaphragm pump (KNF UN035STI/EX) downstream of the FTIR gas cell. The flow rate through the FTIR gas cell was maintained at 7 SLPM using an Alicat MCP-10SLPM-D mass flow controller calibrated for air.

**Table 4:** FTIR probe details per vehicle.

Vehicle Code	Probe Location	Sampling Interval (s)	Sampling Line Length (m)
G-HK-F2	Plume (3.05 m above pan)	5.6	25.6
G-TR-F	Plume (3.05 m above pan)	5.6	25.6
E-HK-S	Car interior (Rear seat)	5.6	27.4
E-CB-S	Car interior (Rear seat)	5.6	27.4
E-TM-S	Car interior (Rear seat)	5.6	27.4
E-FM-S	Car interior (Rear seat)	5.6	27.4
E-FM-B	Car interior (Rear seat)	1.9	29.3
E-TM-B	Car interior (Rear seat)	1.9	29.3
E-FM-SA	Car interior (Rear seat)	1.9	29.3
E-FM-BS	Car interior (Rear seat)	1.9	29.3
E-TM-BS	Car interior (Rear seat)	1.9	29.3

In addition to the FTIR probe, gases from the cabin of the vehicle were collected for particulate fluoride and hydrofluoric acid quantification for a subset of experiments. Each sample was collected using a 6.4 mm (1/4 in.) C276 nickel allow tube. The sample gas was passed through a fluoride filter (SKC225-9031) at a flow rate of 2 L min^-1^, set prior to pilot ignition. The fluoride filter was sent to an external lab for analysis and quantification of particulate fluoride and hydrogen fluoride as per NIOSH 7906. The details of fluoride sampling for various vehicles are listed in Table 5.

**Table 5:** Details of fluoride sampling per vehicle.

Vehicle Code	Probe location	Time sampled (min)	Volume sampled (L)
E-CB-S	Car interior (Rear seat)	62.2	124.43
E-FM-S	Car interior (Rear seat)	37.5	75
E-FM-B	Car interior (Rear seat)	126	253
E-TM-B	Car interior (Rear seat)	101	202
E-FM-SA	Car interior (Rear seat)	55.5	111
E-FM-BS	Car interior (Rear seat)	210.5	421
E-TM-BS	Car interior (Rear seat)	75.5	151

Area air around the vehicles was sampled using active air sampling pumps placed at various positions to allow for characterization of products of combustion with known health concerns. Gas sampling methodologies such as the flow rate, sampling media, and analytical method, were dependent on the targeted gas species and are shown in Table 6.

**Table 6:** Area gas sampling media, flow rates, analytical methods, levels of detection (LODs), and units.

Compound	Sampling Media	Flow Rate (L min^-1^)	Analytical Method	Level of Detection (LOD)	Units
BTEXS	SKC 226-01	0.2	NIOSH 1501	0.5-10*	mg
1,3-Butadiene	SKC 226–09	0.25	NIOSH 1024	1	mg
Aldehydes	SKC 226-117	0.1	OSHA 52/OSHA 68	1-2**	mg
Isocyanates	Asset EZ4-NCO	0.5	ISO 17734	15	ng
Metals	Mixed Cellulose Esters Membrane (MCE)	2.5	NIOSH 7303	1	mg
SO2	SKC 225-9005	1.5	NIOSH 6004	4	mg
HCN	SKC 225-710 \& SKC 226-210	0.2	NIOSH 6010	1	mg
HF	SKC 225-9031	2.0	NIOSH 7903*	1	mg
HCl	SKC 225-9032	0.2-2***	NIOSH 7907	3	mg
PAHs	SKC 226-57	1.0	NIOSH 5528	0.1	mg

*LOD was 0.5, 2, 2, 10, and 4 μg for benzene, toluene, ethylbenzene, xylenes, and styrene, respectively.

**LOD was 1, 2, 2 μg for formaldehyde, acetaldehyde, and acrolein, respectively.

***Flow rate for HCl was 0.2 L min^-1^ for freeburn tests and 2.0 L min^-1^ for tests with firefighting interventions.

Combustion products were analyzed for volatile organic compounds (VOCs), aldehydes, isocyanates, metals, and acid gases. The VOCs assessed included benzene, toluene, ethylbenzene, xylenes, and styrene (collectively referred to as BTEXS), along with 1,3-butadiene. Isocyanates included methyl isocyanate, methylene diphenyl diisocyanate, and phenyl isocyanate. The specific aldehydes examined were acetaldehyde, acrolein, and formaldehyde, all of which are also associated with respiratory and cardiovascular impacts. Metal analysis targeted chromium, cobalt, copper, lead, lithium, manganese, aluminum, nickel, antimony, arsenic, and cadmium. Acid gases measured included hydrogen cyanide (HCN), hydrogen chloride (HCl), and hydrogen fluoride (HF). Particulate fluoride was also analyzed and in a subset of the tests, particulate chloride was analyzed.

Sampling media were positioned at various locations depending on the test. For the freeburn tests, sampling media were placed 3.0 m in front of the vehicle, 4.5 m behind the rear, and near the ceiling 8.3 m above the vehicle. Sampling pumps were housed within a steel enclosure that was thermally shielded and insulated to protect against ambient heat and radiant energy. The pumps and media were connected via tubing to 7.5 cm ceramic tubes that passed through ports in the enclosure. At the 3.0 m and 4.5 m locations, the sampler inlet tubing was oriented horizontally with the openings directed toward the vehicle. For ceiling-level sampling in the plume, the inlet tubing was positioned vertically, with the openings facing upward. For suppression and blanket tests, sampling media were placed 4.5 m behind the rear of the vehicle, near the ceiling 8.3 m above the vehicle, and a subset of samples were carried by the firefighter performing firefighting operations. Pumps were carried in a pack, behind the firefighter’s breathing apparatus, with sampling lines running over the shoulder to the firefighter’s breathing zone.

Sampling pumps (Gilian BDX-II, Sensidyne, St. Petersburg, FL) were calibrated to within ±5% of the target flow rates specified in Table 6. All sampling media were handled according to the respective method outlined in Table 6 and transported to the analytical laboratory on ice, maintaining chain of custody throughout.

The reported mass measurements are the total mass of each compound that was collected throughout the duration of each test (data_airsampling.csv). Not all measurements were collected at all locations. Samples that were not collected are noted as “NA”. In some cases, collected samples did not have values above the level of detection (LOD) for the analytical method. These samples are noted as “<LOD”. Sampling duration is listed as the total number of minutes that the sampling pumps ran (data_airsamplingtime.csv). For pumps carried by firefighters, pumps were started and stopped when the crew entered and exited the fire lab.

### 4.7 Data acquisition

Thermocouple, heat flux gauge, pressure transducer (FTIR), and water flow meter data were recorded at 2 Hz (exp G-HK-F through E-HK-F) and 1 Hz (exp G-HK-F2 through E-TM-BS) using a National Instruments data acquisition system (PXIe-1088 chassis, PXIe-8861 controller, PXIe-4353 temperature modules, and PXIe-4302 voltage modules), and saved in CSV format. Mass data from the platform scale indicators were recorded at 0.5 Hz using Collect+ version 1.2.1.1 (Mettler Toledo) and saved in CSV format. Infrared thermogram sequences were recorded at 30 Hz using ViperVision 4.38.24 (Viper Imaging), and saved in compressed sequence (CSQ) format. The burner mass flow controller output was recorded at 1 Hz using FlowVision version 2.0 software (from Alicat), and saved in CSV format. Data acquisition devices were started simultaneously to ensure that all measurements were time-synced.

### 4.8 Data analysis procedures

#### 4.8.1 Events and time series data

All time series data were shifted such that zero time corresponds to confirmation of burner ignition. Event times provided in the data repository are in global time (format hh:mm:ss). All data in the timeseries data was processed to be delivered in the native units: temperature (°C), heat flux (kW m-^2^), flow rate (gal min^-1^), and mass (kg).

#### 4.8.2 Heat release rate

Mass measurements from the four platform scale indicators were summed to produce the total mass (m) at each time step (i). The mass loss rate (MLR) was then calculated by numerically approximating the derivative of the vehicle mass over time:

$$\text{ML}R_{i}=\frac{m_{i}-m_{i-1}}{\Delta t}$$

where Δt is the time step (2 seconds, per the 0.5 Hz recording frequency). Initial filtering of this mass loss rate was done by replacing mass loss rate points greater than 0.05 kg s^-1^ with a rolling average of the neighboring 30 mass loss rate points. The entire mass loss rate dataset was then smoothed with a 15-point centering rolling average. Any remaining erroneous data from personnel leaning on the pan and load cells during burner ignition was removed manually, times for which are detailed in Table 7.

**Table 7:** Details on erroneous data removed manually from mass data files.

Experiment	Start time (s)	End time (s)	Total (s)
G-HK-F	-80	-4	76
E-CB-F	-3	+183	186
E-NL-F	-101	+41	152
E-HI-F	-5	+19	24
E-TM-F	-48	+62	110
E-FM-F	-31	+63	94
E-HK-F	-	-	-
G-HK-F2	-	-	-
G-TR-F	-	-	-

The heat release rate of the fire was calculated as the product of the computed mass loss rate and an assumed effective heat of combustion of 25 MJ kg^-1^ [5]. This technique is referred to as mass loss calorimetry, and while there is uncertainty associated with the assumption of a constant value of effective heat of combustion over the burning duration of the fire, it has been used in several studies to measure heat release rates of large vehicle fires.

#### 4.8.3 Heat flux

A Schmidt-Boelter heat flux gauge generates a thermoelectric response ($E_{g}$) that is proportional to the net heat flux to the sensing element of the gauge (${{\dot{q}}^{\prime \prime }}_{g}$), which consists of a copper constantan thermopile coated with a high absorptivity spectrally flat paint. The proportionality constant ($C_{g}$) is termed the responsivity. Gauge heat flux was calculated using the following expression:

$${{\dot{q}}^{\prime \prime }}_{g}=\frac{1}{C_{g}}E_{g}$$

The gauges used in this study, including both total heat flux and windowed radiometer gauges, were calibrated by the manufacturer. The absorptivity of all gauges was reported to be 0.94 per the manufacturer’s calibration. Windowed radiometers were calibrated with their sapphire windows in place; accordingly, the calculated gauge heat flux accounts for the transmissivity of the windows.

Infrared thermogram sequences of the driver-side and passenger-side plate sensor arrays were recorded at 30 Hz. These sequences were decimated to 1 Hz, trimmed to exclude the beginning and end of the experiments (prior to the onset of fire spread from the initiating burner and after the fire was effectively extinct), and exported to CSV format (one file per second). The start and end times (relative to burner ignition) of the thermogram sequence are provided in the events file for each experiment. These raw CSV thermogram sequences are available in a separate data repository [6]. The thermogram sequences were then distortion-corrected and cropped to the corresponding areas of the sensing surfaces of the six plate sensors (three per side), resulting in a total of six thermogram sequences per experiment. These processed thermogram sequences were saved as HDF5 binary files, which are provided in the data repository.

Each of the computations were performed using the open-source HFITS program [7]. The data repository contains the measured plate sensor temperature data, as well as the incident radiative heat flux computed from those data. For additional details, the reader is referred to the source material that describes this measurement and analysis procedure [4].

### 4.9 Smoke species composition

The interferograms acquired by the FTIR instrument were processed by Bruker’s OPUS GA software to give transmission spectra. The instrument acquired a background transmission spectrum by averaging 120 interferogram scans before the start of data acquisition. The background corrected transmission spectra, starting at pilot ignition, are included with this article.

## Limitations

Missing Data:

• Scale failure in 2 tests (G-HK-F2 and G-TR-f)
• Electrical failure in E-FM-S (short data outage)
• TC measurements post suppression vulnerable to damage

Skipped data:

• Mass change was NaN’d during initial burner placement (described)

Limited size of dataset:

• Field heat flux and mass loss only measured for free burns, as the presence of the instruments would interfere with firefighting operations in the remaining experiments.
• FTIR was only acquired in a subset of experiments.

## Ethics Statement

The authors have read and follow the ethical requirements for publication in Data in Brief and confirm that the current work does not involve human subjects, animal experiments, or any data collected from social media platforms.

## CRediT Author Statement

**Nathaniel G. Sauer:** Conceptualization, methodology, software, investigation, data collection, data curation, writing, original draft preparation. **Matthew J. DiDomizio:** Conceptualization, methodology, software, investigation, data collection, data curation, writing, original draft preparation. **Richard M. Kesler:** Conceptualization, methodology, investigation, data collection, data curation, writing, original draft preparation. **Shruti Ghanekar:** Conceptualization, methodology, software, investigation, data collection, data curation, writing, original draft preparation. **Parham Dehghani:** Conceptualization, methodology, software, data curation, writing, original draft preparation. **Gavin P. Horn:** Conceptualization, methodology, investigation, data collection, data curation, supervision. **Adam Barowy:** Conceptualization, methodology, investigation, resources, data collection, writing, supervision, original draft preparation.

## Data Availability

figshareMeasurement data from full-scale fire experiments of battery electric vehicles and internal combustion engine vehicles (Original data)

## References

[1] Sauer N.G., DiDomizio M.J., Kesler R.M., Ghanekar S., Dehghani P., Horn G.P., Barowy A. (2025). Measurement data from full-scale fire experiments of battery electric vehicles and internal combustion engine vehicles. UL Res. Inst. Dataset.

[2] Kerber S. (2012). Analysis of changing residential fire dynamics and its implications on firefighter operational time frames. Fire Technol..

[3] Kang S., Kwon M., Choi J.Y., Choi S. (2023). Full-scale fire testing of battery electric vehicles. Appl. Energy.

[4] DiDomizio M.J., Dehghani P. (2025). Measuring two-dimensional heat flux using a plate sensor and infrared thermography. MethodsX.

[5] Hodges J.L., Salvi U., Kapahi A. (2024). Design fire scenarios for hazard assessment of modern battery electric and internal combustion engine passenger vehicles. Fire Saf. J..

[6] Sauer N.G., DiDomizio M.J., Kesler R.M., Ghanekar S., Dehghani P., Horn G.P., Barowy A. (2025). Plate sensor temperature data from full-scale fire experiments of battery electric vehicles and internal combustion engine vehicles. UL Res. Inst., Dataset.

[7] P. Dehghani and M.J. DiDomizio, 2024, “HFITS: An analysis tool for calculating heat flux to planar surfaces using infrared thermography”, SoftwareX, vol. 28, pp. 101934, 10.1016/j.softx.2024.101934.

